# Is metadata of articles about COVID-19 enough for multilabel topic classification task?

**DOI:** 10.1093/database/baae106

**Published:** 2024-10-21

**Authors:** Shuo Xu, Yuefu Zhang, Liang Chen, Xin An

**Affiliations:** College of Economics and Management, Beijing University of Technology, No. 100 PingLeYuan, Chaoyang District, Beijing 100124, P.R. China; College of Economics and Management, Beijing University of Technology, No. 100 PingLeYuan, Chaoyang District, Beijing 100124, P.R. China; Institute of Scientific and Technical Information of China, No. 15 Fuxing Road, Haidian District, Beijing 100038, P.R. China; School of Economics and Management, Beijing Forestry University, No. 35 Qinghua East Road, Haidian District, Beijing 100083, P.R. China

## Abstract

The ever-increasing volume of COVID-19-related articles presents a significant challenge for the manual curation and multilabel topic classification of LitCovid. For this purpose, a novel multilabel topic classification framework is developed in this study, which considers both the correlation and imbalance of topic labels, while empowering the pretrained model. With the help of this framework, this study devotes to answering the following question: Do full texts, MeSH (Medical Subject Heading), and biological entities of articles about COVID-19 encode more discriminative information than metadata (title, abstract, keyword, and journal name)? From extensive experiments on our enriched version of the *BC7-LitCovid* corpus and *Hallmarks of Cancer* corpus, the following conclusions can be drawn. Our framework demonstrates superior performance and robustness. The metadata of scientific publications about COVID-19 carries valuable information for multilabel topic classification. Compared to biological entities, full texts and MeSH can further enhance the performance of our framework for multilabel topic classification, but the improved performance is very limited.

**Database URL**: https://github.com/pzczxs/Enriched-BC7-LitCovid

## Introduction

The COVID-19 pandemic continues to expand globally, resulting in a significant surge in the number of related articles, estimated to increase by 10 000 per month [1]. In response, the LitCovid dataset [1–3] was established from PubMed database to collect COVID-19-related articles, amassing over 370 000 articles to date. The dataset is updated daily and the articles are organized into seven research topics: “treatment,” “mechanism,” “prevention,” “case report,” “diagnosis,” “transmission,” and “epidemic forecasting.” Although automated methods for annotating the resulting topics have been developed, the performance in term of F_1_ score is not satisfactory [1]. For this purpose, the LitCovid Track for COVID-19 literature topic annotations in BioCreative VII called for a community effort [4, 5].

This challenge is actually a multilabel classification task that aims at assigning one or more topic labels to each article. In this challenge, a considerable amount of fields (such as titles, abstracts, keywords, and journal names) have been made available. Though many other fields, including full texts, biological entities, and MeSH (Medical Subject Heading), are also available via PubMed/LitCovid, these fields are seldom utilized by participating teams [4]. We argue that main reasons are 2-fold: (i) it is not trivial to directly retrieve these fields from PubMed/LitCovid, since many fields are actually missed due to copyright restrictions (see subsection “Data enrichment” for more detail); (ii) many pretrained models [6–8] limit the processed sequences to be no longer than 512 tokens due to their memory and computational requirements.

In addition, similar to many multilabel classification problems in real-world applications, two prominent characteristics [9] are also observed from topic annotations in the LitCovid dataset [4]: “label correlation” and “label imbalance.” However, only two teams [10, 11] considered label imbalance and one team [12] took label correlation into consideration in their solutions. In our opinion, the main reason may be that it is not easy to directly incorporate these features into pretrained models due to time constraints. Therefore, we identify the following research questions:

1. Do full texts, biological entities, and MeSH encode more discriminative information than metadata (such as title, abstract, keyword, and journal name)?
2. How to empower pretrained model-based multilabel classification approach by considering label correlation and imbalance?

The remaining sections of this article are organized as follows. After a comprehensive review on multilabel classification, methods provided in Sections “Related work” and “Datasets” describe in more detail how to further collect full texts, biological entities, and MeSH on the basis of *BC7-LitCovid* corpus [4]. Then, a detailed framework for multilabel topic classification is presented in Section “Methodology.” Section “Experimental results and discussions” offers an exhaustive account of the experiment, coupled with a corresponding discussion. The final section concludes this study with a summary of our main contributions and several limitations.

## Related work

Many multilabel learning methods have been proposed in the literature. Generally speaking, these methods can be divided into three groups: problem transformation methods, algorithm adaptation methods, and ensemble methods [13]. In the subsequent subsections, this study briefly reviews these three categories of methods one by one.

### Problem transformation approaches

The problem transformation methods aim to tackle multilabel problem by transforming it to one or more single-label problems, which are easier to be solved. This kind of method can be further grouped into four main strategies: Binary Relevance (BR) [14], ClassifierChain (CC) [15], Pair-Wise (PW) [16], and Label Powerset (LP). The BR strategy treats a multilabel problem as multiple separate binary classification problems (one label as positive class and the others as negative one) [14]. It is apparent that the BR strategy does not leverage the correlations between labels. To further exploit the potential correlations between labels and enhance model performance, the label correlations can be modelled using a chain structure or other control structures such as Bayesian networks [17]. The PW strategy views a multilabel problem as multiple binary classification problems (one label as positive class and another as negative one) and then adopts the majority voting mechanism to determine the final prediction label, such as Calibrate Label Ranking (CLR) [18]. The LP-set strategy regards each label combination as a category, thus transforming a multilabel classification problem into a multiclass classification problem. It is not difficult to see that this transformation results in a very large label combination space [19].

### Algorithm adaptation approaches

The algorithm adaptation methods devote to solving the multilabel problem by adapting popular learning methods to accommodate multilabel datasets directly. The following state-of-the-art methods are usually adapted: Decision Tree, Tree-Based Boosting, Lazy Learning, Support Vector Machine (SVM), Neural Network, and so on.

Clare *et al*. [20] introduced the information gain in the Decision Tree to represent the discriminative ability of features to all labels. The Adaboost is an iterative algorithm, which constantly updates the weight of the estimated sequence through a weak classifier, and obtains the final prediction label by weight voting after a specific number of iteration rounds, including Adaboost.MH and Adaboost.MR [21]. The Lazy Learning algorithms usually take the search of *k*-nearest examples as a first step, and then calculate the distance to make the final decision of the labels, just like Multi-Label *k*-Nearest Neighbor Algorithm (ML*k*NN) [22]. The basic idea of SVM is to adopt the “maximum margin” strategy, minimizing the ranking loss by defining a set of linear classifiers and introducing “kernel trick” to deal with the nonlinear classification problem, such as rank-SVM [23] and Multi-Label Least-Squares Support Vector Machine (ML^2^S-SVM) [9].

The neural network-based algorithms are extensively utilized to handle multilabel classification problems by assigning multiple labels at the output layer [24]. Several neural network architectures, including Convolutional Neural Networks (TextCNN) [25], Bidirectional Long Short-Term Memory (Bi-LSTM) [26], PatentNet [27], Bioformer [28], and Bidirectional Encoder Representation from Transformers (BERT) [6], fall into this category. In general, most of these neural network-based methods share a similar framework comprising two main modules: a neural network and a label predictor. For instance, Xu *et al*. [29] utilized four deep learning models, such as TextCNN [25] and FastText [30], to address the multilabel classification problem for COVID-19. Their approach achieved superior performance compared to the baseline in terms of label-based and instance-based F1 scores. Xu *et al*. [31] comprehensively compared the performance of seven multilabel classification algorithms on three real-world patent and publication datasets, and found that neural network-based models showed obvious superiority on small-scale datasets with more balanced document-label distribution, but traditional machine learning-based models work better on the large-scale datasets with more imbalanced document-label distribution.

### Ensembles approaches

The ensemble methods seek to learn the classifier by cutting and replacing the observed subsets, or by analyzing various label sorts in the chain. The Ensembles of Classifier Chains (ECC), the Ensembles of Pruned Sets (EPS), the ensembles of neural network-based algorithms, and the Random *k*-LabelSets (RA*k*EL) are the resulting representatives of the ensemble methods.

The ECC is a technique that selects the best labels by summarizing the votes of each label when using the Classifier Chains (CC) [15, 32, 33] as a base classifier and standard bagging scheme. The EPS algorithm combines pruned sets in an ensemble scheme for the fast building speed of pruned sets [34]. Meanwhile, the integration of neural network-based algorithms aims to amalgamate various deep learning techniques to resolve the problem of multilabel topic classification. Gu *et al*. [10] and Lin *et al*. [11] used BERT and transformer-based methods, respectively, to deal with the COVID-19 multilabel topic classification problem.

On the other hand, the RA*k*EL utilizes an ensemble of Label Powerset classifiers, where each *k*-labelset is trained as a multilabel classifier. Note that each subset of an initial set of labels (such as a set of seven topic labels mentioned in Section “Introduction”) is called a “labelset,” and a *k*-labelset is a labelset of the size *k*. During the prediction phase, the algorithm employs a voting mechanism, enabling RA*k*EL to effectively take into account the correlations between labels and make predictions for unseen label combinations. Moreover, this approach is able to effectively deal with the challenge of encountering a large number of classes in the LP strategy [35].

It has been shown that the ensemble approaches usually outperform problem transformation approaches and algorithm adaptation approaches [10, 11, 36]. Therefore, this study utilizes the RA*k*EL to model the correlations among labels, in which SVM [37] is taken as a base classifier for modeling label imbalance. In addition, due to powerful representation capability of deep learning techniques, a pretrained neural network model is used to generate the resulting representation vector of each article in this work.

## Dataset

The *BC7-LitCovid* corpus was released by the LitCovid Track challenge organizers [4], which consisted of 33 699 records of COVID-19-related articles. This corpus was further partitioned into train set (24 960), development set (6239), and test set (2500), as shown in Table 1. Note that only metadata (such as title, abstract, keyword, and journal name) is involved in this corpus. To answer our first research question in Section “Introduction,” we enrich this corpus with full texts, biological entities, and MeSH. It is noteworthy that Lin *et al*. [11] and Bagherzadeh *et al*. [38] also used MeSH information to participate in the LitCovid Track for COVID-19 literature topic annotations in BioCreative VII.

**Table 1.** Statistic description of the *BC7-LitCovid* corpus

Field	Train	Development	Test	All
Title	24 960 (100%)	6239 (100%)	2500 (100%)	33 699 (100%)
Abstract	24 919 (99.8%)	6223 (99.8%)	2489 (99.6%)	33 631 (99.8%)
Keywords	18 968 (76.0%)	4754 (76.2%)	2056 (82.2%)	25 778 (76.5%)
Journal name	24 960 (100%)	6239 (100%)	2500 (100%)	33 699 (100%)
*Full texts*	22 551 (90.34%)	5599 (89.74%)	2413 (96.52%)	30 563 (90.7%)
*Biological entities*	23 252 (93.16%)	5804 (93.02%)	2219 (88.76%)	31 275 (92.8%)
*MeSH*	19 450 (77.92%)	4853 (77.78%)	1428 (57.12%)	25 731 (76.4%)

### Label distribution

**Figure 1.**
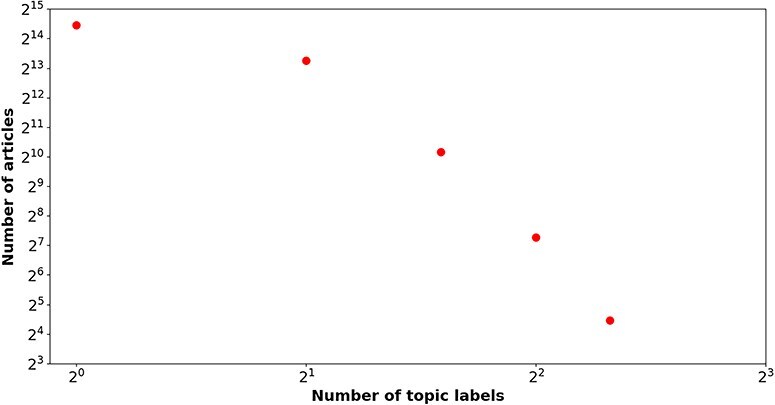
The number of topic labels (*x*-axis) attached to articles (*y*-axis) in the *BC7-LitCovid* corpus，in which both axes are shown on a log scale.

Table 2 delineates the label distribution among the train, development, and test sets in the *BC7-LitCovid* corpus. An obvious imbalance can be readily observed in term of the number of articles of each topic label. The topic label “Prevention” is responsible for 31.87% articles, while the topic label “Epidemic Forecasting” accounts for only 1.89% ones. In this way, the number of articles for “Prevention” and “Epidemic Forecasting” is about 17:1. Figure 1 illustrates the relation between the number of labels and that of articles. A power law-like distribution is evident from near linear pattern in Fig. 1. That is to say, more than four topic labels are simultaneously assigned to a few articles (176 in total), but the vast majority of articles (22 547 in total) have only one topic label. For example, a literature review (PMID = “32623267”) is attached with five topic labels (“Treatment,” “Mechanism,” “Prevention,” “Diagnosis,” and “Transmission”).

**Table 2.** The label distribution in the *BC7-LitCovid* corpus

Label	Train	Development	Test	All
Treatment	8718 (25.46%)	2207 (25.95%)	1035 (28.62%)	11 960 (25.79%)
Mechanism	4439 (12.96%)	1073 (12.61%)	567 (15.68%)	6079 (13.11%)
Prevention	11 102 (32.42%)	2750 (32.33%)	926 (25.61%)	14 778 (31.87%)
Case report	2063 (6.02%)	482 (5.67%)	197 (5.45%)	2742 (5.91%)
Diagnosis	6193 (18.08%)	1546 (18.18%)	722 (19.97%)	8461 (18.25%)
Transmission	1088 (3.18%)	256 (3.01%)	128 (3.54%)	1472 (3.17%)
Epidemic forecasting	645 (1.88%)	192 (2.26%)	41 (1.13%)	878 (1.89%)

**Figure 2.**
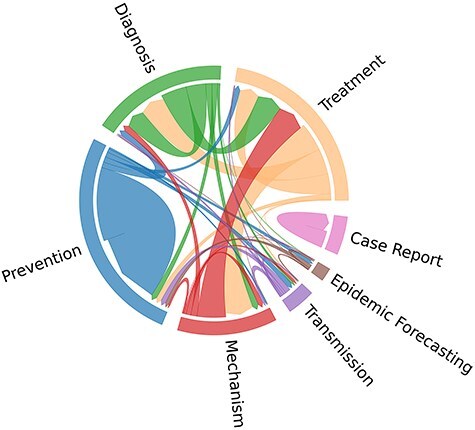
Cooccurrence distribution of topic labels in the *BC7-LitCovid* corpus.

In Fig. 2, the cooccurrence patterns among topic labels in the *BC7-Litvoid* corpus are further elucidated. From Fig. 2, it is observed that “Treatment” and “Mechanism,” and “Treatment” and “Diagnosis,” are usually assigned to one same article. In contrast, “Case Report” topic label appears to be isolated, implying that it is not associated with any other topics. These observations imply the existence of correlations among the designated topic labels. Notably, the train set, development set, and test set exhibit a similar distribution pattern across different labels. Moreover, no hierarchical structure among seven labels is observed.

### Data enrichment

**Figure 3.**
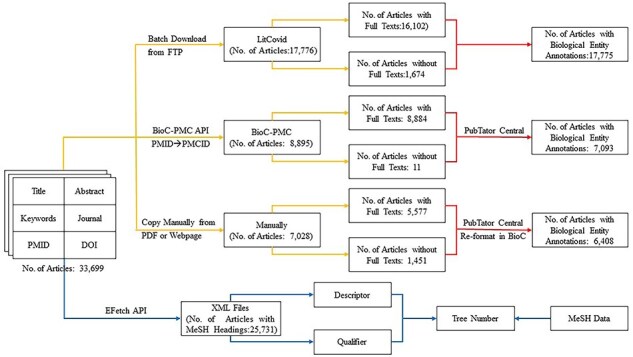
Procedure of data enrichment for the *BC7-LitCovid* corpus.

As mentioned earlier, only title, abstract, keywords, journal name, PMID (PubMed Identifier), and DOI (Digital Object Identifier) [39] are available in the *BC7-LitCovid* corpus [1]. To check whether full texts, biological entities, and MeSH can further improve the performance of multilabel topic classification task, the *BC7-LitCovid* corpus is enriched with the procedure in Fig. 3. The specific detail for these types of information is described one by one at length in the following subsections.

#### Full texts

Just as its name implies, the *BC7-LitCovid* corpus is actually a subset of the *LitCovid* dataset [1]. In this way, one can directly download the *LitCovid* dataset in buck from LitCovid FTP (https://ftp.ncbi.nlm.nih.gov/pub/lu/LitCovid/), and then extract the resulting full texts from it by matching the PMID of each article. The second way is to fetch the full texts in the BioC-XML format [40] with BioC-PMC API (https://www.ncbi.nlm.nih.gov/research/bionlp/APIs/BioC-PMC/) after mapping PMID to PMCID (PubMed Central Identifier). Note that not all fetched articles are attached with the resulting full texts. To eliminate these articles, the XML files with the size <10 kb are checked manually one by one, and then removed if the resulting full texts cannot be found. As for the other articles, the full texts are copied manually if the corresponding webpages or PDF files can be found through PMID, PMC, or DOI. It is worth mentioning that the articles in non-English language or out of subscription by Beijing University of Technology are excluded from collecting full texts in this study. In the end, 30 563 articles with full texts, accounting for 90.69% ones in the *BC7-LitCovid* corpus, can be obtained, as shown in Fig. 3.

**Figure 4.**
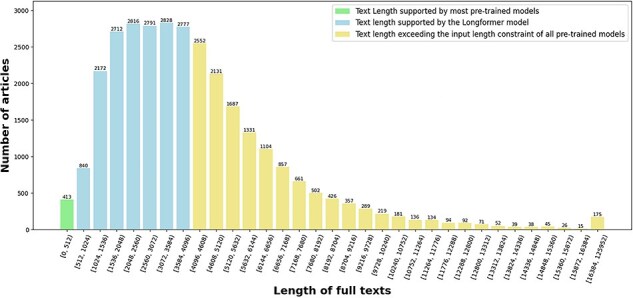
The length distribution of full texts in terms of the number of tokens in our enriched version of the *BC7-LitCovid* corpus.

The length distribution of full texts in term of the number of tokens is depicted in Fig. 4. One can see that only 413 articles (1.35%) have a length less than 512 tokens, which is the length limit of many pretrained models [6–8], and 17 349 articles (56.76%) have a length less than 4096 tokens, which is the length limit of the Longformer pretrained model [41]. About 13 214 articles (43.24%) have more than 4096 tokens in their full texts. This observation validates our speculation in Section “Introduction” that the vast majority of the full texts exceed the input length constraint of many pretrained models.

#### Biological entity annotations

This study considers five types of biological entities: “cell line,” “chemical,” “disease,” “gene,” and “species.” To recognize biological entity mentions [42], similar to full texts, three ways as illustrated in Fig. 3 are involved. (i) The biological entity annotations can be directly parsed from the *LitCovid* dataset downloaded in the last subsection. (ii) With the web service provided by PubTator Central [43], one can readily retrieve biological entities by submitting the texts in the BioC-XML format. (iii) As for collected manually articles, the resulting texts are reformatted to the BioC format [40] before utilizing the PubTator Central service. It is noteworthy that due to unknown reasons, the PubTator Central cannot annotate any biological entities from several articles, though these articles indeed mention many relevant entities. As for this issue, we have consulted the PutTator Central via email, but no responses are obtained until now. Therefore, these articles are assumed not to mention any biological entities at all.

**Figure 5.**
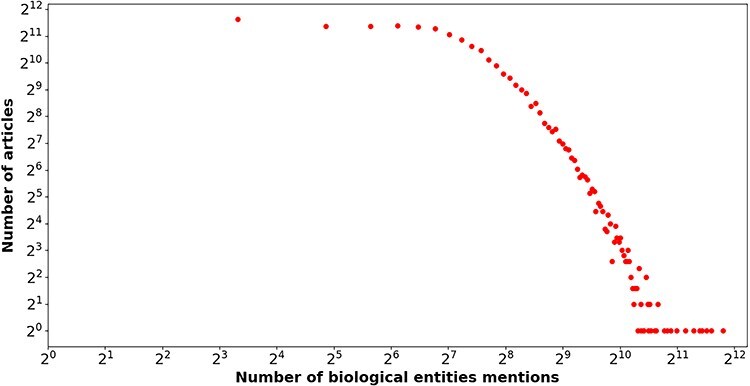
The number of biological entities (*x*-axis) mentioned in articles (*y*-axis) in our enriched version of the *BC7-LitCovid* corpus, in which both axes are shown on a log scale.

In the end, the number of articles with biological entity annotations is 31 275, which accounts for 92.81% of the total articles. The number of biological entity mentions and unique entities are 4 945 619 and 186 890, respectively, and the distribution of number of biological entity mentions over entity types is reported in Table 3. Figure 5 shows the relation between the number of entity mentions and that of articles. Similar to Fig. 1, a power law-like distribution is again observed from near-linear pattern in Fig. 5. To say it in another way, the great majority of biological entities are mentioned by a few of articles, but a few of biological entities are mentioned by a large number of articles. For instance, the article with PMID= “32325980” mentioned 3574 biological entities. The average number of biological entity mentions per article is 158.13.

**Table 3.** Distribution of number of biological entity mentions over entity types

Entity type	No. of mentions	Entity type	No. of mentions
Gene	681 850 (13.79%)	Species	1 849 267 (37.39%)
Disease	1 830 970 (37.02%)	Cell line	17 052 (0.34%)
Chemical	566 480 (11.45%)		
Σ	4 945 619		

#### MeSH headings

**Figure 6.**
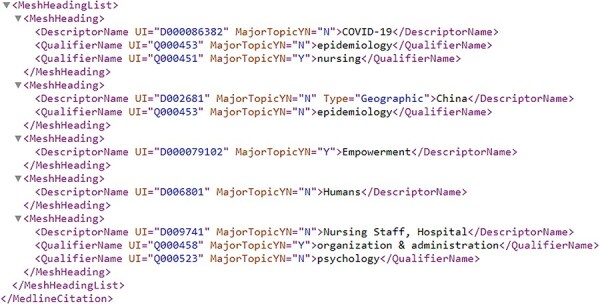
MeSH heading list for the article with PMID = “32259325”.

**Figure A1.**
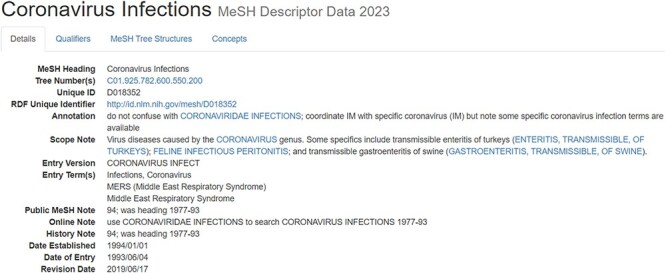
MeSH Descriptor with UI = “D018352”.

**Figure A2.**
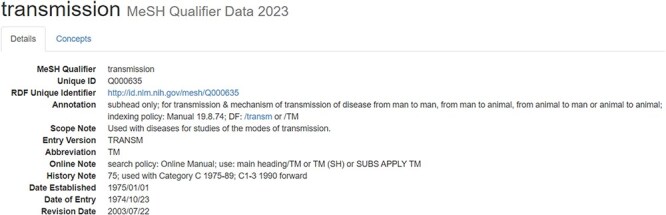
MeSH Qualifier with UI = “Q000635”.

Compared to full texts and biological entity annotations, the MeSH headings are a little complicated. For ease of understanding, the article with PMID = “32259325” is taken as an example, whose MeSH headings are illustrated in Fig. 6. From Fig. 6, it is ready to see that multiple “descriptors” are assigned to this article, and each “descriptor” may be constrained by more than one “qualifier.” More details on the “descriptor” with UI (Unique ID) = “D018352” and the “qualifier” with UI = “Q000635” is shown in Fig. A1 and Fig. A2, respectively, in Appendix.

**Figure 7.**
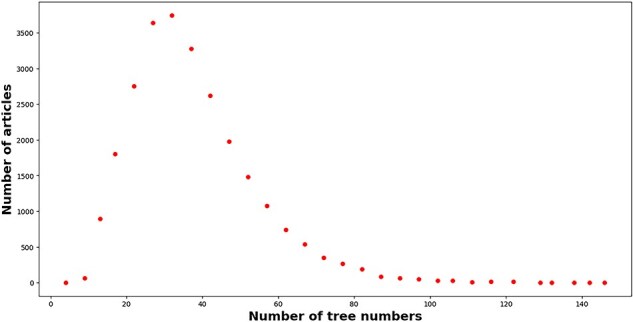
The number of tree numbers (*x*-axis) attached to articles (*y*-axis) in our enriched version of the *BC7-LitCovid* corpus.

As a matter of fact, MeSH headings including “descriptors” and “qualifiers” are positioned to different hierarchies in a tree-like classification scheme (https://meshb.nlm.nih.gov/treeView). However, the name and UI of each MeSH headings cannot carry any semantics about the resulting hierarchy. To leverage the hierarchy information of each MeSH headings, this work maps UI to the resulting “tree number(s)” with the help of MeSH data in the XML format (https://www.nlm.nih.gov/databases/download/mesh.html) after retrieving MeSH headings of each article with EFetch API (https://www.ncbi.nlm.nih.gov/books/NBK25499/#chapter4.EFetch). In the end, the number of articles with MeSH headings is 25 731, which is only 76.36% of the total articles. The number of tree number mentions and unique tree numbers is 957 003 and 18 849, respectively. As a matter of fact, several MeSH heading extractors have been developed in the literature, such as MTI [44], pyMeSHSim [45], and DeepMeSH [46]. For simplicity, we do not supplement the missing MeSH Heading information. Figure 7 displays the distribution of tree numbers within articles. A right-skewed distribution emerges. A large proportion of articles (95.73%) mentioned tree numbers within the range of 10–70. On average, each article mentions 37 192 tree numbers. Only a small proportion of articles (0.42%) mentioned more than 100 tree numbers. For example, 146 tree numbers are mentioned in the article with PMID = “32279418.”

## Methodology

**Figure 8.**
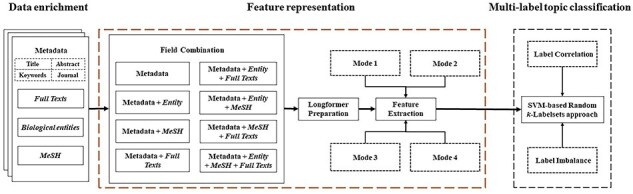
Research framework for multilabel topic classification.

For the purpose of multilabel topic classification for COVID-19 literature, as shown in Fig. 8, our research framework mainly consists of three modules: data enrichment, feature representation, and multilabel topic classification. The data enrichment module collects full texts, biological entities, and MeSH. For more elaborate and detailed description, we refer the readers to Subsection “Data enrichment.” Then, each combination of fields is represented by a vector through feature extraction on the basis of Longformer model [41]. In this module, the limitation of sequence length in most pretrained models is loosened, so that our framework can benefit from full texts. Finally, the feature vectors with the resulting topic labels are fed to the random *k*-labelsets method [47] with SVM as a base classifier (SVM-based random *k*-labelsets for short) for multilabel topic classification. In this module, the random *k*-labelsets method is utilized to model the high-order correlations among topic labels [36], and SVM is to deal with label imbalance by setting different weights for positive and negative instances [9, 37]. This framework further elucidates our second research problem: how to enhance pretrained models for multilabel classification by taking into account label correlation and imbalance. In the following subsections, feature representation and multilabel topic classification are described at length.

### Feature representation

The feature representation addresses the problem of finding the most compact and informative set of features to enhance the performance of machine-learning algorithm in a focal task [48]. In this module, the neural network model is utilized for feature representation. Specifically, we use the second-generation of pretrained models to yield a contextual word embedding for each article. Compared to the context-independent word embeddings produced by the first-generation pretrained models (such as Word2vec [49] and GloVe [50]), the contextual word embeddings are more advantageous as they take the context of each word into consideration.

**Figure 9.**
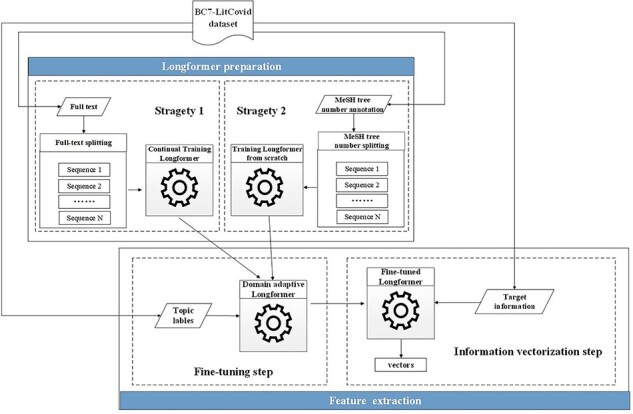
The procedure of feature representation on the basis of the Longformer model.

The most representatives of the second generation of pretrained models are BERT [6] and its variants. Many successful applications based on these models can be found in the literature [7]. But their memory and computational requirements grow quadratically with sequence length, making it infeasible (or very expensive) to process the sequences longer than 512 tokens in our case (cf. green histograms in Fig. 4). Hence, the Longformer model [41], which is able to scale linearly with the sequence length, is utilized here for our feature representation. Though, maximal input length for the Longformer model is 4096 tokens, which is still far below the length of many full texts in the enriched version of the *BC7-LitCovid* corpus (cf. blue histograms in Fig. 4). For this purpose, the whole procedure for feature representation in this study includes two components: Longformer preparation and feature extraction, as shown in Fig. 9. The former makes the Longformer model more adaptive to our case. The latter further customizes the Longformer model to the task of multilabel topic classification and transforms the information from our enriched version of the *BC7-LitCovid* corpus into the resulting vectors. The specific detail of each component will be described as follows.

#### Longformer preparation

Many pretrained models are usually trained on general-domain corpora, such as BookCorpus [51], English Wikipedia, and CommonCrawl News dataset [52]. However, when dealing with highly domain-specific texts, like COVID-19-related articles in this work, the textual distinction between general domain and specific one will severely jeopardize the classification performance. There are two strategies to alleviate this situation as follows [1]. A neural network model is retrained from scratch with a domain-specific corpus. It is obvious that the cost is extremely expensive in terms of data volume and computation cost [2]. One can start from a pretrained model on general domain corpus, and then continue to train this model for domain adoption.

As a matter of fact, these two strategies are used simultaneously here. More specifically, as for metadata, full texts, and biological entities, continual training strategy is adopted since compared with general corpora mentioned earlier, the amount of *BC7-LitCovid* corpus is limited. The procedure of corpus preparation for continual training strategy is illustrated in Algorithm 1.

**Table:** 

**Algorithm 1**: The procedure of corpus preparation for continual training strategy				
**Input**: *BC7-LitCovid* corpus				
**Output**: Prepared corpus for continual training strategy				
1:	Let *Q* be the output list			
2:	**for** each paper *p* in the *BC7-LitCovid* corpus **do**			
3:		Let cursor position *cp*: = 0, *cache*: = null		
4:		**while** *cp* is not the end of *p***do**		
5:			*np*:= *p*.getNextPassage(*cp*)	
6:			*cp*:= *cp* + *np*.length	
7:			*np_tokens*: **= **convertPassageToTokens(*np*)	
8:			**if** *np_tokens*.length*+ cache*.length *≥ *4096 **then**	
9:				*cp*:= *cp*—*np*.length
10:				*Q*.append(*cache*)
11:				*cache*: = null
12:			**Else**	
13:				*cache*: = *cache* + *np_tokens*
14:			**end if**	
15:		**end while**		
16:	**end for**			
17:	**return** *Q*			

**Figure 10.**
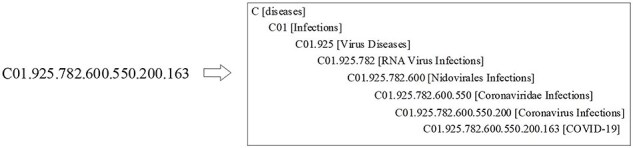
A structure example of the MeSH tree number.

**Figure 11.**
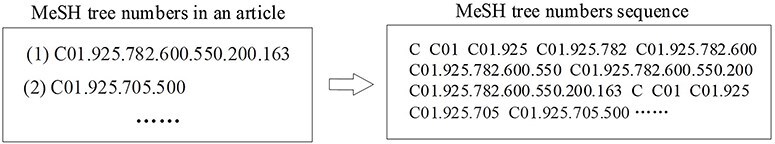
The procedure of generating a sequence from MeSH tree numbers in an article.

As for MeSH headings, this study treats the resulting tree numbers in our enriched version of the *BC7-LitCovid* corpus as a special corpus and separately trains a MeSH tree numbers oriented Longformer from scratch. Let’s take the article in Fig. 6 as an example. The resulting MeSH tree numbers (such as C01.925.782.600.550.200.163 and C01.925.705.500) can be retrieved via EFetch API. Since MeSH tree number follows a tree-like structure, as shown in Fig. 10, in which the higher hierarchy has the broader meaning and the lower hierarchy has the specific meaning, all MeSH tree numbers in different hierarchies can be achieved and concatenated into a sequence to describe the article in multiple hierarchies in Fig. 11. By assembling these sequences into a corpus, it is convenient to train a MeSH tree number-oriented Longformer from scratch. Intuitively, this strategy suffers from insufficient data, but in practice it is an effective way for feature extraction from MeSH tree numbers. We argue that the main reason is tree numbers follow a tree-like structure, and to our knowledge no pretrained models can accommodate this structure until now.

#### Feature extraction

**Figure 12.**
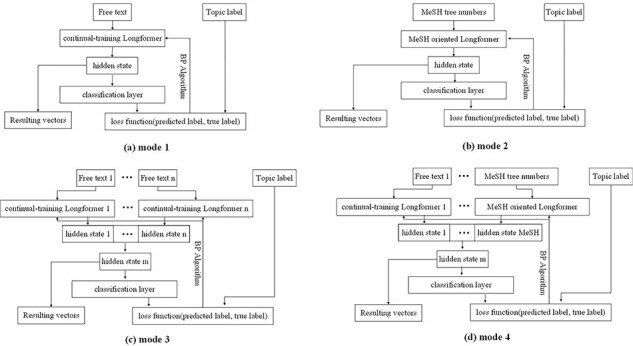
Four modes for fine-tuning Longformer models.

This component utilizes the Longformer model to transform the information in our enriched version of the *BC7-LitCovid* corpus, namely target information, to the resulting vectors, viz. feature extraction. In more detail, a subset of samples are randomly picked from the enriched version of the *BC7-LitCovid* corpus, then the target information and their labels are combined as a training set to customize the Longformer model for multilabel topic classification task. This is so-called fine-tuning in the literature. With the fine-tuned Longformer model, one can transform each article into the corresponding vector for multilabel topic classification task. The basic fine-tuning process is shown in Fig. 12a, where the target information, namely free text in our case, is put into the Longformer model, and represented by a vector. Then, the vector is passed through the classification layer and the predicted labels are output. With the help of back propagation (BP) algorithm, the predicted labels will approximate the true labels. This enables the Longformer model to adapt to the multilabel topic classification task.

According to the composition of target information, four fine-tuning modes are provided in Fig. 12. The mode 1 is for single textual information with short length, such as biological entities. The mode 2 is for MeSH tree numbers, which is also of short length. The mode 3 is for textual information with length surpassing input limit of Longformer model, or for the combination of multiple textual information, like biological entities and metadata. In this case, after textual information is divided into several spans, these spans are transformed into different vectors by multiple Longformer instances, and then these vectors are concatenated into one vector. Compared to mode 3, a MeSH tree numbers oriented Longformer in previous subsection is added in mode 4.

### Multilabel topic classification

Table 2 reports the distribution of the original labels in the BC7-LitCovid corpus, and Table A1 in Appendix shows the distribution of the labelsets. It is apparent that both the labels and their corresponding sets demonstrate a markedly imbalanced distribution. For example, only one article is annotated simultaneously by “treatment,” “mechanism,” and “epidemic forecasting.” This will deteriorate the multilabel topic classification performance. Therefore, to mitigate the skewness degree of distribution of class values as well as to model the higher-order label correlations among labels (cf. Figure 2), Tsoumakas *et al*. [35] developed the random *k*-LabelSets (RA*k*EL) approach. In more detail, an initial set of labels is broken into a number of the labelsets of size *k* (i.e. *k*-labelset) in the first place, and then the LP approach [47] is employed to train a resulting multilabel classifier for each lableset. To determine the resulting multilabel of an unseen instance, the decisions from all LP classifiers are gathered and combined.

Two distinct strategies for constructing the *k-labelsets* are involved in the RA*k*EL approach as follows: (i) the strategy of “disjoint” labelsets (“disjoint” strategy for short), and (ii) the strategy of “overlapping” labelsets (“overlapping” strategy for short). It has been shown that the overlapping strategy outperforms the disjoint one, since the aggregation of multiple predictions for each label via voting is able to correct potential errors to some extent [35]. Therefore, the overlapping strategy is utilized here.

The distribution of labelsets with *k* = 2 is reported in Table A2 in Appendix. It is obvious that a less skewed distribution of class values than that in the original labelsets can be obtained. Though, the skewness degree of distribution of class values is still very serious. Hence, the SVM is utilized as the base classifier in the LP approach [47] to deal with the problem of skewed distribution by assigning different weights to positive and negative instances [9, 37]. On this basis, further improvement in the skewness degree of the class value distribution can be achieved by adjusting the output shape of the decision function.

## Experimental results and discussions

### Evaluation measures

Since each article is usually attached with multiple topic labels, performance evaluation in multi-label classification is much more complicated than that in classic single-label classification. For this study, we assess the performance of our framework through instance-based and label-based (macro, micro) F1 measures, which are formally defined as follows.


(1) $$\begin{array}{*{20}{c}}
{F{1_{{\mathrm{micro }}}} = 2 \cdot \frac{{{\mathrm{ Precision}}{{\ }_{{\mathrm{micro }}}} \cdot {\mathrm{ Recall}}{{\ }_{{\mathrm{micro }}}}}}{{{\mathrm{ Precision}}{{\ }_{{\mathrm{micro }}}} + {\mathrm{ Recall}}{{\ }_{{\mathrm{micro }}}}}}{\ }}
\end{array}$$

(2) $$\begin{array}{*{20}{c}}
{F{1_{{\mathrm{macro }}}} = 2 \cdot \frac{{{\mathrm{ Precision}}{{\ }_{{\mathrm{macro }}}} \cdot {\mathrm{ Recall}}{{\ }_{{\mathrm{macro }}}}}}{{{\mathrm{ Precision}}{{\ }_{{\mathrm{macro }}}} + {\mathrm{ Recall}}{{\ }_{{\mathrm{macro }}}}}}{\ }}
\end{array}$$

(3) $$F{1_{{\mathrm{instance\,}}}} = \frac{1}{{\mathrm{N}}}\sum\nolimits_{i = 1}^n {2 \cdot \frac{{{\mathrm{\,Precision}}{{\mathrm{\,}}_{{\mathrm{i\,}}}} \cdot {\mathrm{\,Recall}}{{\mathrm{\,}}_{{\mathrm{i\,}}}}}}{{{\mathrm{\,Precision}}{{\mathrm{\,}}_{{\mathrm{i\,}}}} + {\mathrm{\,Recall}}{{\mathrm{\,}}_{{\mathrm{i\,}}}}}}} $$

Here, “Precision” and “Recall” are frequently employed in multilabel classification measures [53], and they are calculated based on the number of true positives, true negatives, false positives, and false negatives at both the instance-based and label-based levels.

### Experimental setup

The selection of the penalty term ($C$), width coefficient of the kernel function ($\gamma $), and the kernel type is crucial for the SVM classifier. We follow the recommendation in [54] to establish the range for $C$ and $\gamma $($C$ ∈ {2^−5^, 2^–3^,…, 2^15^}, $\gamma $ ∈ {2^−15^, 2^–13^,…, 2^3^}). We employ a grid search strategy to determine the optimal parameter combination. The radial basis function (RBF) is adopted as our kernel function. In addition, if one views each *k*-labelsets as a category, a multi-category classification problem is involved in the SVM classifier. The one-vs-one (OVO) strategy is utilized for multi-category classification in the SVM classifier. The main reasons are 2-fold: (i) the imbalance mentioned in subsection “Multi-label topic classification” can be further alleviated and (ii) the OVO strategy outperforms the other strategies in most cases [55].

**Figure A3.**
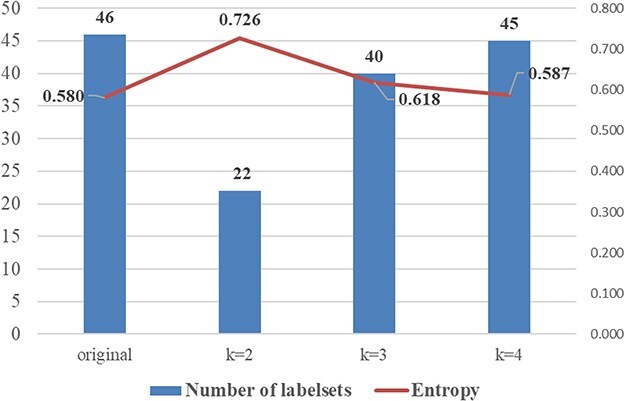
The number of unique labelsets and normalized entropy over the desired size of each partition (*k*). The normalized entropy is the cumulative logarithms of probability distributions for each labelset within articles. An increase in the normalized entropy value signifies more imbalanced instances for each labelset.

As for the RA*k*EL algorithm, there are two crucial parameters: the desired size of each partition (*k*), which determines the size of label combinations, and the number of trained sub-classifiers. Tsoumakas *et al*. [35] recommended to configuring a relatively small value for *k*. In consideration of the varying quantities of labelsets for different values of *k* (cf. Table A1 in Appendix), as well as the normalized entropy values illustrated in Fig. A3 in Appendix, we opt to set the value of *k* to 2. Increasing the number of trained sub-classifiers results in an enhancement in performance, but it is accompanied by a corresponding increase in computational cost. Following the recommendation in [35], the number of trained sub-classifiers is fixed to double of the number of unique topic labels (i.e. 14).

This study uses the training set of the *BC7-LitCovid* corpus as the training set and the development set as the validation set to determine five optimal parameter combinations for each field combination, as shown in Table 4. Using the optimal parameter combinations for each field combination, we train our model on the combined training and development sets and then predict the resulting topic labels of each article in the test set.

**Table 4.** Optimal parameter combinations for the SVM classifier on our enriched version of the *BC7-LitCovid* corpus

	Optimal parameters ($C,\gamma $)				
Field combination	Run 1	Run 2	Run 3	Run 4	Run 5
Metadata	2^5^, 2^–5^	2^7^, 2^–7^	2^9^, 2^–7^	2^9^, 2^–11^	2^15^, 2^–15^
Metadata + entity	2^11^, 2^–7^	2^13^, 2^–9^	2^13^, 2^–11^	2^15^, 2^–11^	2^15^, 2^–13^
Metadata + MeSH	2^7^, 2^–7^	2^11^, 2^–7^	2^11^, 2^–9^	2^13^, 2^–11^	2^15^, 2^–13^
Metadata + full texts	2^9^, 2^–7^	2^11^, 2^–7^	2^13^, 2^–9^	2^15^, 2^–11^	2^15^, 2^–13^
Metadata + entity + full texts	2^5^, 2^–5^	2^7^, 2^–5^	2^9^, 2^–5^	2^11^, 2^–7^	2^13^, 2^–11^
Metadata + entity + MeSH	2^7^, 2^–7^	2^9^, 2^–7^	2^9^, 2^–9^	2^11^, 2^–11^	2^13^, 2^–11^
Metadata + MeSH + full texts	2^7^, 2^–5^	2^9^, 2^–7^	2^11^, 2^–9^	2^13^, 2^–9^	2^13^, 2^–11^
Metadata + entity + MeSH + full texts	2^9^, 2^–7^	2^11^, 2^–7^	2^11^, 2^–9^	2^13^, 2^–11^	2^15^, 2^–13^

### Performance on different field combinations

To better answer the first research question posed in this paper, we conduct a comprehensive analysis to evaluate the performance of different field combinations. This allows us to assess whether full texts, biological entities, and MeSH terms encode more discriminative information than metadata.

From Table 5, one can see that our approach achieves remarkable performance using only metadata, with a maximum macro-F1 of 86.13%, micro-F1 of 90.28%, and instance-based F1 of 91.11%, respectively. The further inclusion of biological entities does not yield a significant performance enhancement, and even lowers the performance only on metadata. In contrast, the inclusion of MeSH headings results in a slight overall performance improvement, which is consistent with the findings from other research teams [11, 38]. Additionally, the inclusion of full texts leads to a 1.3% increase in term of macro F1, but not in an improvement in term of micro F1 and instance-based F1. When compared to the substantial effort invested in enriching full texts (cf. subsection “Data enrichment”), only 1.3% enhancement in term of macro F1 does not seem to be satisfactory. This implies that the metadata of articles about COVID-19 encodes enough discriminative information for multilabel topic classification. This observation is in line with that in Gu *et al*. [10] and Lin *et al*. [11].

**Table 5.** Performance comparison on different field combinations

	Run 1 (%)			Run 2 (%)			Run 3 (%)			Run 4 (%)			Run 5 (%)		
	MiF	MaF	InF	MiF	MaF	InF	MiF	MaF	InF	MiF	MaF	InF	MiF	MaF	InF
1	90.02	85.40	90.95	89.98	85.61	90.90	89.98	85.81	90.82	90.25	85.74	**91.20**	**90.28**	**86.13**	91.11
2	89.80	85.79	90.67	90.00	85.78	**90.82**	89.71	85.73	90.60	**90.04**	**86.11**	90.74	89.92	85.99	90.74
3	**90.31**	**86.37**	**91.11**	89.94	86.07	90.67	90.29	86.26	91.08	90.21	86.21	90.95	90.06	86.11	90.79
4	**90.25**	87.09	**91.11**	90.18	**87.43**	90.87	90.09	87.06	90.91	90.14	87.16	90.99	90.05	86.68	91.00
5	**90.25**	**86.58**	**91.09**	90.22	86.49	91.00	89.91	86.20	90.65	90.05	86.41	90.87	90.08	86.27	90.93
6	89.79	85.96	90.74	89.68	85.63	90.63	**89.87**	86.02	**90.80**	89.85	**86.13**	90.80	89.64	85.84	90.55
7	89.94	85.75	90.71	90.18	86.19	90.93	90.25	86.37	91.01	90.09	85.83	90.79	**90.30**	**86.76**	**91.04**
8	90.13	85.67	90.89	89.87	85.54	90.64	**90.32**	**85.95**	**90.99**	90.27	85.65	91.02	90.24	85.66	91.00

The bolded cells indicate the best performance over different runs for each field combination. For clarity, the field combinations are listed as follows. 1: Metadata, 2: metadata + entity, 3: metadata + MeSH, 4: metadata + full texts, 5: metadata + entity + full texts, 6: metadata + entity + MeSH, 7: metadata + MeSH + full texts, 8: metadata + entity + MeSH + full texts. In addition, MiF, MaF, and InF denote micro-F1, macro-F1, and instance-based F1, respectively.

### Performance on the largest sub-corpus

As illustrated in Table 1, a significant number of field information remains incomplete. To further validate the robustness of our findings, we extract all articles with no missed field information as the largest sub-corpus. Following the partition criterion in the *BC7-LitCovid* corpus, this study generates a training set (16 003 articles), a development set (3960 articles), and a test set (1221 articles). With the optimal parameter combination in Table 4, the performance on various field combinations is reported Table 6.

**Table 6.** Performance comparison on different field combinations of the largest sub-corpus

	Run1 (%)			Run2 (%)			Run3 (%)			Run4 (%)			Run5		
	MiF	MaF	InF	MiF	MaF	InF	MiF	MaF	InF	MiF	MaF	InF	MiF	MaF	InF
1	**90.31**	**84.99**	**91.30**	90.24	84.61	91.18	89.94	84.27	90.89	89.99	84.89	91.13	90.01	84.75	91.05
2	90.11	85.64	90.73	89.98	85.39	90.70	90.18	85.46	90.91	90.04	85.76	90.79	**90.40**	**85.92**	**91.07**
3	**90.15**	84.42	91.01	89.93	84.86	90.71	89.86	84.45	90.73	89.99	84.53	90.78	90.13	**84.87**	**91.03**
4	90.40	86.55	**91.31**	90.36	86.57	91.09	**90.44**	**87.00**	91.26	90.34	86.57	91.09	90.33	86.49	**91.31**
5	90.53	85.67	91.25	**90.78**	**86.64**	**91.45**	90.35	84.66	91.04	90.22	85.39	90.82	90.17	85.78	90.74
6	90.17	85.93	91.16	89.75	85.34	90.61	**90.30**	**86.68**	**91.21**	90.06	**86.26**	90.96	89.79	85.53	90.66
7	90.43	85.34	91.18	90.62	86.14	91.27	90.73	86.03	91.46	90.66	85.73	91.32	**90.80**	**86.51**	**91.50**
8	**90.66**	**85.32**	91.39	90.08	84.05	90.85	90.51	84.59	91.30	90.52	84.42	91.25	90.60	84.97	**91.40**

The bolded cells indicate the best performance over different runs for each field combination. For clarity, the field combinations are listed as follows. 1: Metadata, 2: metadata + entity, 3: metadata + MeSH, 4: metadata + full texts, 5: metadata + entity + full texts, 6: metadata + entity + MeSH, 7: metadata + MeSH + full texts, 8: metadata + entity + MeSH + full texts. In addition, MiF, MaF, and InF denote micro-F1, macro-F1, and instance-based F1, respectively.

From Table 6, similar conclusions on the entire corpus can be drawn again. That is to say, the inclusion of biological entities or MeSH headings does not yield significant performance improvements. The inclusion of full texts results in a substantial 2.01% increase in term of macro F1 score, and relatively small improvements of 0.13% and 0.01% in terms of micro and instance-based F1 scores, respectively.

As we progressively incorporate various fields into the analysis, several interesting discoveries can be obtained. In comparison to metadata, both micro- and instance-based F1 on the field combination of metadata and MeSH experience a decline. Simultaneously, the performance on the field combination of metadata and full texts shows a slight improvement in terms of micro and instance-based F1 scores. In contrast, the field combination of metadata, MeSH, and full texts exhibits slightly larger improvements in terms of micro- and instance-based F1 compared to the field combination of metadata and MeSH, with increases of 0.65% and 0.47%, respectively. Similar trends can also be observed in the field combinations involving biological entities and full texts. These observations suggest that full texts can serve as an alternative to biological entities. After all, the full texts mention these biological entities.

### Ablation study

Our research framework, founded on Longformer’s text vector representations, considers imbalance and label correlation to improve multilabel topic classification performance. To illustrate the effectiveness of different components within the proposed framework, we perform an ablation study on the validation set. As depicted in Tables 5 and Table 6, the utilization of metadata alone produces commendable results. Therefore, only metadata is used for the ablation experiments to streamline the experimental comparisons.

The detail of the ablation experiments is presented in Table 7, with the best scores highlighted in bold. The ablation experiments are divided into four variants. The first utilizes the Longformer model to extract metadata features, which are then fed into the Sigmoid activation function for multilabel topic classification, evaluated through the label probability matrix. The others, based on vectors extracted by the Longformer model, employ machine-learning models for multi-label topic classification. Specifically, the second employs the Binary Relevance classification strategy [14], disregarding label correlation and imbalance. The third addresses label imbalance by assigning different weights to labels but does not consider label correlation. The fourth employs the RA*k*EL approach to account for label correlation. The experimental results indicate that considering label imbalance and label correlation can enhance classification performance to some extent, with our method achieving superior classification performance.

**Table 7.** Ablation experimental results on the metadata of *BC7-LitCovid* corpus

Experiment name	MiF (%)	MaF (%)	InF (%)
Longformer with sigmoid	49.06	25.55	51.90
Unbalanced, independent labels	90.07	85.93	90.88
Balanced, independent labels	**90.30**	86.00	**91.23**
Unbalanced, correlated labels	90.14	85.90	90.97
Proposed method	90.25	**87.43**	91.11

The bolded cells indicate the best performance over different experiments.

### Ensemble of different experimental results

In order to enhance the performance and facilitate comparisons with other research teams, we conduct ensemble learning on the results of entire corpus. Specifically, we compute the average probability of topic labels for each article by utilizing the topic label matrices obtained from the eight field combinations. For field combinations 1–8, the optimal parameters (C, γ) are determined as follows: [2^15^, 2^–15^], [2^15^, 2^–11^], [2^7^, 2^–7^], [2^9^, 2^–7^], [2^5^, 2^–5^], [2^11^,2^–11^], [2^13^,2^–11^], and [2^11^, 2^–9^].

The assessment results of our ensemble experiment are presented in Table 8. In comparison to the baseline outcomes, our top-performing model demonstrates a 5.67% increase in terms of micro F1, a 9.3% increase in term of macro F1, and a 4.9% increase in term of instance-based F1. These findings demonstrate the competitiveness of our framework in Fig. 8. The performance of our approach is very close to the third-ranking method in the LitCovid Track for COVID-19 literature topic annotations in BioCreative VII (cf. Table 8). On closer examination, the following two factors contribute better performance for top 3 teams. (i) These studies have undergone extensive pretraining on a substantial corpus of COVID-19 articles [10, 57], whereas we conduct pretraining exclusively on the *BC7-LitCovid* corpus. (ii) Multiple pretrained models (including homogenous and heterogeneous models) [10, 11] are integrated, while we just employ the Longformer model and a homogenous SVM-based random *k*-labelsets classifier.

**Table 8.** Comparison with top 3 team submission results

	MiF (%)	MaF (%)	InF (%)
Baseline [56]	85.56	78.47	87.01
Top 1 [10]	93.03	90.94	94.77
Top 2 [11]	92.00	88.81	93.92
Top 3 [57]	91.81	88.75	93.94
ClaC [38]	88	86	–
Our approach	91.23	87.80	91.93

For clarity, the Top 2 team and the CLaC team also utilized MeSH headings.

### Performance validation on the HoC corpus

Despite the limited availability and small scale of existing multi-label classification datasets in the biomedical field, coupled with the complexity associated with full texts data collection, we extend our research framework to the HoC corpus [58]. This extension aims to rigorously evaluate the robustness and applicability of our multilabel classification research framework.

This corpus comprises 1580 PubMed abstracts, meticulously annotated on the basis of scientific evidence pertaining to the 10 currently recognized HoC. Du *et al*. [56] further partitioned this corpus into train, development, and validation sets. Similar to the *BC7-LitCovid* corpus, we supplement the corpus with full texts, biological entities, and MeSH information. Notably, due to unavailability of PubTator API, biological entities are recognized with the Hunflair tool [59]. Detailed information is provided in Table 9.

**Table 9.** Statistic description of the HoC corpus

Field	Train	Development	Test	All
Title	1108 (100%)	157 (100%)	315 (100%)	1580 (100%)
Abstract	1104 (99.96%)	153 (97.45%)	313 (99.34%)	1570 (99.37%)
Keywords	562 (50.72%)	80 (51.00%)	154 (48.89%)	796 (50.38%)
Journal name	1108 (100%)	157 (100%)	315 (100%)	1580 (100%)
Full texts	1055 (95.22%)	145 (92.36%)	294 (93.33%)	1494 (94.56%)
Biological entities	1108 (100%)	157 (100%)	315 (100%)	1580 (100%)
MeSH	1056 (95.31%)	149 (94.90%)	294 (93.33%)	1499 (94.87%)

This study employs identical methods for feature extraction, parameter optimization, and ensemble learning, with the final experimental results presented in Table 10. For more details, please refer to the Table A3 and Table A4 in Appendix. Compared to the baseline method, the performance of metadata in this study improved instance-based F1 by 4.75%, and after ensemble learning, the performance increased by 6.51%, demonstrating the robustness and generalizability of the model. Additionally, consistent with the aforementioned conclusions, the inclusion of full texts leads to a 0.44% increase in term of macro-F1, but does not improve micro-F1 and instance-based F1. Given that substantial efforts are invested in enriching full texts (cf. subsection “Data enrichment”), only a 0.44% enhancement in term of macro-F1 appears unsatisfactory. However, contrary to the above conclusion, the inclusion of biological entities and MeSH results in a greater improvement in multilabel topic classification performance. This discrepancy may be attributed to the following reasons:

**Table 10.** Performance validation on the Hoc corpus

	MiF (%)	MaF (%)	InF (%)
Baseline [56]	–	–	82.90
Metadata	85.80	85.80	87.65
Metadata + entity	87.08	87.80	88.66
Metadata + MeSH	86.36	86.72	88.16
Metadata + full texts	85.77	86.24	87.48
Metadata + entity + full texts	87.08	87.03	88.93
Metadata + entity + MeSH	84.79	84.48	86.04
Metadata + MeSH + full texts	83.14	83.46	85.31
Metadata + entity + MeSH + full texts	85.47	85.71	87.66
Ensemble	87.43	88.11	89.41

(a) The articles in the HoC corpus were typically published before 2011, whereas COVID-19 articles were often published from 2019 onwards, which may reflect differences in writing styles.

(b) The PubTator [43] and Hunflair [59] tools are utilized respectively to recognize biological entities from the *BC7-LitCovid* and *HoC* corpora. The performance for biological entity recognization may further affect multilabel topic classification performance.

## Conclusions

The substantial increase in the volume of Covid-19-related publications has presented significant challenges for multilabel topic assignment. Though many automated approaches for assigning the resulting topics have been developed in the literature, full texts, biological entities, and MeSH are seldom taken into consideration due to copyright and text length restrictions. Furthermore, two prominent characteristics (“label correlation” and “label imbalance”) in the LitCovid dataset have not been paid enough attention in previous pretrained model-based approach for multilabel topic classification.

For this purpose, a novel framework for multilabel topic classification is proposed in this study, which empowers pretrained models by taking label correlation and imbalance into account. In more detail, a pretrained model scaling linearly with the sequence length (viz. Longformer model) is utilized here for feature representation, and the RA*k*EL with SVM as a base classifier for dealing with label correlation and imbalance. Extensive experimental results on various feature combinations in our enriched version of the *BC7-LitCovid* corpus indicate that the metadata of scientific publications about COVID-19 carries valuable information for multilabel topic classification. Compared to biological entities, full texts and MeSH can further enhance the performance of our framework for multilabel topic classification, but the improved performance is very limited.

By ensemble of experimental results on different field combinations, the performance of our framework is very close to that of the third-ranking method in the LitCovid Track for COVID-19 literature topic annotations in BioCreative VII. Additionally, for the multilabel topic classification task in the HoC corpus, our framework demonstrates a 6.51% improvement in terms of instance-based F1 score over the same baseline. This confirms the effectiveness of our proposed framework. Despite the usefulness of our framework, this study is subject to the following limitations. Due to the scarcity of multilabel classification datasets and the challenges associated with collecting full-text data, our analysis is constrained to the *BC7-LitCovid* corpus and the HoC corpus. Therefore, further scientific validation of our methodology is required in future research. Furthermore, we aim to incorporate a more extensive dataset, such as the LitCovid dataset [1–3] and Cord-19 dataset [60, 61], to pretrain the Longformer model. Additionally, exploring the application of models capable of handling infinite context [62] within our framework presents a promising direction for future work.

## Data Availability

The enriched version of BC7-LitCovid corpus can be freely accessed at: https://github.com/pzczxs/Enriched-BC7-LitCovid.

## Appendix

**Table A1.** Distribution of original labelsets

Labelset	No. of articles	Labelset	No. of articles	Labelset	No. of articles	Labelset	No. of articles
3	12 040	2, 5	279	1, 2, 5, 6	35	5, 7	6
1, 2	3902	7	265	1, 5, 6	33	1, 7	5
1	3265	1, 3, 5	214	2, 5, 6	31	1, 3, 5, 7	3
5	3239	6	172	2, 3, 6	30	3, 5, 7	2
1, 5	3213	2, 6	162	1, 3, 5, 6	26	1, 2, 7	1
4	2740	5, 6	133	1, 2, 3, 5, 6	23	1, 3, 7	1
2	808	3, 5, 6	83	2, 3, 5	20	2, 3, 7	1
3, 6	574	3, 6, 7	64	6, 7	19	2, 6, 7	1
1, 2, 5	552	1, 2, 3, 5	60	2, 3, 5, 6	15	1, 2, 6, 7	1
3, 7	508	1, 2, 3	58	1, 3, 6	14	2, 3, 5, 7	1
1, 3	487	2, 3	47	1, 2, 3, 6	12		
3, 5	484	1, 2, 6	36	1, 6	8		

To make it clear, topic label corresponding to the first column are listed as follows. 1: “treatment,” 2: “mechanism,” 3: “prevention,” 4: “case report,” 5: “diagnosis,” 6: “transmission,” and 7: “epidemic forecasting.”

**Table A2.** Distribution of labelsets with *k* = 2

Labelset	No. of articles	Labelset	No. of articles	Labelset	No. of articles	Labelset	No. of articles
3	12 040	2, 5	1016	5, 6	379	6, 7	85
1, 2	4680	3, 5	931	2, 6	346	5, 7	12
1, 5	4159	1, 3	898	2, 3	267	1, 7	11
1	3265	3, 6	841	7	265	2, 7	5
5	3239	2	808	1, 6	188		
4	2740	3, 7	580	6	172		

To make it clear, topic label corresponding to the first column are listed as follows. 1: “treatment,” 2: “mechanism,” 3: “prevention,” 4: “case report,” 5: “diagnosis,” 6: “transmission,” and 7: “epidemic forecasting.”

**Table A3.** Optimal parameter combinations for the SVM classifier on our enriched version of the Hallmarks of Cancer corpus

	Optimal parameters ($C,\gamma $)				
Field combination	Run 1	Run 2	Run 3	Run 4	Run 5
Metadata	2^–3^, 2^–9^	2–^1^, 2^–11^	2^1^, 2^–11^	2^3^, 2^–15^	2^5^, 2^–13^
Metadata + Entity	2^1^, 2^–5^	2^5^, 2^–13^	2^7^, 2^–15^	2^7^, 2^–11^	2^9^, 2^–7^
Metadata + MeSH	2^–1^, 2^–7^	2^1^, 2^–9^	2^3^, 2^–9^	2^5^, 2^–15^	2^7^, 2^–15^
Metadata + full texts	2^1^, 2^–5^	2^5^, 2^–9^	2^7^, 2^–11^	2^9^, 2^–15^	2^11^, 2^–15^
Metadata + entity + full texts	2^3^, 2^–9^	2^7^, 2^–11^	2^9^, 2^–15^	2^11^, 2^–15^	2^13^, 2^–15^
Metadata + entity + MeSH	2^1^, 2^–7^	2^3^, 2^–9^	2^5^, 2^–7^	2^7^, 2^–5^	2^9^, 2^–5^
Metadata + MeSH + full texts	2^1^, 2^–7^	2^5^, 2^–7^	2^5^, 2^–5^	2^7^, 2^–5^	2^9^, 2^–5^
Metadata + entity + MeSH + full texts	2^–1^, 2^–5^	2^1^, 2^–5^	2^5^, 2^–5^	2^9^, 2^–7^	2^13^, 2^–13^

**Table A4.** Performance comparison on different field combinations (HoC corpus)

	Run 1 (%)			Run 2 (%)			Run 3 (%)			Run 4 (%)			Run 5 (%)		
	MiF	MaF	InF	MiF	MaF	InF	MiF	MaF	InF	MiF	MaF	InF	MiF	MaF	InF
1	85.42	85.73	87.43	85.57	86.01	87.50	85.57	85.48	87.59	85.57	**86.41**	87.52	**85.80**	85.80	**87.65**
2	**87.08**	**87.80**	**88.66**	86.63	87.19	88.28	86.22	86.92	87.92	86.49	87.19	87.99	85.11	85.67	86.51
3	86.25	86.37	88.00	86.27	86.42	88.01	85.51	85.51	87.21	86.34	86.30	87.95	**86.36**	**86.72**	**88.16**
4	85.17	85.51	86.96	84.82	85.59	86.76	85.24	85.98	87.12	**85.77**	86.24	**87.48**	85.65	**86.46**	87.43
5	86.50	86.42	88.18	**87.08**	87.03	**88.93**	86.41	86.17	88.38	86.94	86.66	88.67	87.00	**87.09**	88.73
6	**84.79**	**84.48**	**86.04**	84.41	84.34	85.72	84.09	84.20	85.43	83.86	83.75	84.92	84.39	84.22	85.77
7	**83.14**	83.46	**85.31**	82.70	**83.60**	84.87	82.32	83.17	84.49	81.78	83.21	83.84	81.26	82.57	83.35
8	**85.63**	85.36	87.57	85.47	**85.71**	**87.66**	84.12	84.62	86.35	83.91	84.60	85.70	84.43	84.85	86.43

The bolded cells indicate the best performance over different runs for each field combination. For clarity, the field combinations are listed as follows. 1: Metadata, 2: Metadata + Entity, 3: Metadata + MeSH, 4: Metadata + Full Texts, 5: Metadata + Entity + Full Texts, 6: Metadata + Entity + MeSH, 7: Metadata + MeSH + Full Texts, 8: Metadata + Entity + MeSH + Full Texts. In addition, MiF, MaF, and InF denote micro-F1, macro-F1, and instance-based F1 respectively.

## References

[1] Chen Q , Allot A, Lu Z. LitCovid: an open database of COVID-19 literature. *Nucleic Acids Res* 2021;49:D1534–D1540. doi: 10.1093/nar/gkaa952 33166392 PMC7778958

[2] Chen Q , Allot A, Lu Z. Keep up with the latest coronavirus research. *Nature* 2020;579:193–94. doi: 10.1038/d41586-020-00694-1 32157233

[3] Chen Q , Allot A, Leaman R et al. LitCovid in 2022: an information resource for the COVID-19 literature. *Nucleic Acids Res* 2023;51:D1512–D1518. doi: 10.1093/nar/gkac1005 36350613 PMC9825538

[4] Chen Q , Allot A, Leaman R et al. Multi-label classification for biomedical literature: an overview of the BioCreative VII LitCovid Track for COVID-19 literature topic annotations. *Database* 2022:baac069. doi: 10.1093/database/baac069 PMC9428574 36043400

[5] Chen Q , Du J, Allot A et al. LitMC-BERT: transformer-based multi-label classification of biomedical literature with an application on COVID-19 literature curation. *IEEE/ACM Trans Comput Biol Bioinform* 2022;19:2584–95. doi: 10.1109/TCBB.2022.3173562 35536809 PMC9647722

[6] Devlin J , Chang MW, Lee K et al. Bert: pre-training of deep bidirectional transformers for language understanding. *arXiv preprint, arXiv:1810.04805*. 2018.

[7] Lee J , Yoon W, Kim S et al. BioBERT: a pre-trained biomedical language representation model for biomedical text mining. *Bioinformatics* 2020;36:1234–40. doi: 10.1093/bioinformatics/btz682 31501885 PMC7703786

[8] Gu Y , Tinn R, Cheng H et al. Domain-specific language model pretraining for biomedical natural language processing. *ACM Trans Comput Healthc* 2021;3:1–23. doi: 10.1145/3458754

[9] Xu S , An X. ML^2^S-SVM: multi-label least-squares support vector machine classifiers. *Electron Libr* 2019;37:1040–58. doi: 10.1108/EL-09-2019-0207

[10] Gu J , Chersoni E, Wang X et al. LitCovid ensemble learning for COVID-19 multi-label classification. *Database* 2022:baac103. doi: 10.1093/database/baac103 PMC9693804 36426767

[11] Lin SJ , Yeh WC, Chiu YW et al. A BERT-based ensemble learning approach for the BioCreative VII challenges: full-text chemical identification and multi-label classification in PubMed articles. *Database* 2022:baac056. doi: 10.1093/database/baac056 PMC9290865 35849027

[12] Tang W , Wang J, Zhang H et al. Team DUT914 at BioCreative VII LitCovid Track: A BioBERT-based feature enhancement approach. In: *Proceedings of the BioCreative VII Challenge Evaluation Workshop*, pp. 292–294. USA: BioCreative, Cecilia Arighi, University of Delaware, 2021.

[13] Madjarov G , Kocev D, Gjorgjevikj D et al. An extensive experimental comparison of methods for multi-label learning. *Pattern Recognit* 2012;45:3084–104. doi: 10.1016/j.patcog.2012.03.004

[14] Li T , and Ogihara M. Detecting emotion in music. In: *Proceedings of the 4th International Conference on Music Information Retrieval*. Baltimore, MD: The International Society for Music Information Retrieval, 2003.

[15] Read J , Pfahringer B, Holmes G et al. Classifier chains for multi-label classification. *Mach Learn* 2011;85:333–59. doi: 10.1007/s10994-011-5256-5

[16] Fürnkranz J , and Hüllermeier E. Pairwise preference learning and ranking. In: *Proceedings of European Conference on Machine Learning*. Berlin and Heidelberg: Springer, pp. 145–56, 2003.

[17] Zhang ML , and Zhang K. Multi-label learning by exploiting label dependency. In: *Proceedings of the 16th ACM SIGKDD International Conference on Knowledge Discovery and Data Mining*. New York: Association for Computing Machinery, pp. 999–1008, 2010.

[18] Fürnkranz J , Hüllermeier E, Loza Mencía E et al. Multilabel classification via calibrated label ranking. *Mach Learn* 2008;73:133–53. doi: 10.1007/s10994-008-5064-8

[19] Read J , Martino L, Olmos PM et al. Scalable multi-output label prediction: from classifier chains to classifier trellises. *Pattern Recognit* 2015;48:2096–109. doi: 10.1016/j.patcog.2015.01.004

[20] Clare A , and King RD. Knowledge discovery in multi-label phenotype data. In: *Proceedings of European Conference on Principles of Data Mining and Knowledge discovery*. Berlin, Heidelberg: Springer, pp. 42–53, 2001.

[21] Schapire RE . A brief introduction to boosting. In: *Proceedings of the 16th International Joint Conference on Artificial Intelligence*, Vol. 99, pp. 1401–06. San Francisco, CA: Morgan Kaufmann Publishers Inc., 1999.

[22] Zhang ML , Zhou ZH. ML-KNN: a lazy learning approach to multi-label learning. *Pattern Recognit* 2007;40:2038–48. doi: 10.1016/j.patcog.2006.12.019

[23] Elisseeff A , and Weston J. A kernel method for multi-labelled classification. In: *Proceedings of the 14th International Conference on Neural Information Processing Systems: Natural and Synthetic*. Cambridge, MA: MIT Press, pp. 681–7, 2001.

[24] Zhang ML , Zhou ZH. Multilabel neural networks with applications to functional genomics and text categorization. *IEEE Trans Knowl Data Eng* 2006;18:1338–51. doi: 10.1109/TKDE.2006.162

[25] Kim Y . Convolutional neural networks for sentence classification. *arXiv preprint arXiv:1408.5882*. 2014.

[26] Zhou P , Shi W, Tian J et al. Attention-based bidirectional long short-term memory networks for relation classification. In: *Proceedings of the 54th Annual Meeting of the Association for Computational Linguistics*. Berlin: Association for Computational Linguistics, Vol. 2, pp. 207–12, 2016.

[27] Haghighian Roudsari A , Afshar J, Lee W et al. PatentNet: multi-label classification of patent documents using deep learning based language understanding. *Scientometrics* 2022;127:207–31.

[28] Fang L , Chen Q, Wei CH et al. Bioformer: an efficient transformer language model for biomedical text mining. *arXiv preprint, arXiv:2302.01588*. 2023.

[29] Xu S , Zhang Y, and An X. Team BJUT-BJFU at BioCreative VII LitCovid Track: A deep learning based method for multi-label topic classification in COVID-19 literature. In: *Proceedings of the BioCreative VII Challenge Evaluation Workshop*. USA: BioCreative, Cecilia Arighi, University of Delaware, pp. 275–77, 2021.

[30] Joulin A , Grave E, Bojanowski P et al. Fasttext. zip: compressing text classification models. *arXiv preprint, arXiv*:1612.03651. 2016.

[31] Xu S , Zhang Y, An X et al. Performance evaluation of seven multi-label classification methods on real-world patent and publication datasets. *J Data Inf Sci* 2024;9:81–103.

[32] Yu Z , Hao H, Zhang W et al. A classifier chain algorithm with k-means for multi-label classification on clouds. *J Signal Process Syst* 2017;86:337–46. doi: 10.1007/s11265-016-1137-2

[33] Freitas Rocha V , Varejão FM, and Segatto MEV. Ensemble of classifier chains and decision templates for multi-label classification. *Knowl Inf Syst* 2022;64:643–63.

[34] Read J , Pfahringer B, and Holmes G. Multi-label classification using ensembles of pruned sets. In: *Proceedings of the 2008 eighth IEEE International Conference on Data Mining*. pp. 995–1000. Washington, DC: IEEE, 2008.

[35] Tsoumakas G , Katakis I, Vlahavas I. Random k-labelsets for multilabel classification. *IEEE Trans Knowl Data Eng* 2011;23:1079–89. doi: 10.1109/TKDE.2010.164

[36] Zhang ML , Zhou ZH. A review on multi-label learning algorithms. *IEEE Trans Knowl Data Eng* 2013;26:1819–37. doi: 10.1109/TKDE.2013.39

[37] Xu S , Ma F, and Tao L. Learn from the information contained in the false splice sites as well as in the true splice sites using SVM. *Proceedings of International Conference on Intelligent Systems and Knowledge Engineering 2007*. pp. 65–71. Chengdu: Atlantis Press, 2007.

[38] Bagherzadeh P , and Bergler S. CLaC at BioCreative VII LitCovid Track: Independent modules for multi-label classification of Covid articles. In: *Proceedings of the BioCreative VII Challenge Evaluation Workshop*, Cecilia Arighi, University of Delaware. pp. 278–82. USA: BioCreative, 2021.

[39] Xu S , Hao L, An X et al. Types of DOI errors of cited references in web of science with a cleaning method. *Scientometrics* 2019;120:1427–37. doi: 10.1007/s11192-019-03162-4

[40] Comeau DC , Islamaj Doğan R, Ciccarese P et al. BioC: a minimalist approach to interoperability for biomedical text processing. *Database* 2013:bat064. doi: 10.1093/database/bat064 PMC3889917 24048470

[41] Beltagy I , Peters ME, Cohan A. Longformer: the long-document transformer. *arXiv preprint, arXiv:2004.05150*. 2020.

[42] Xu S , An X, Zhu L et al. A CRF-based system for recognizing chemical entity mentions (CEMs) in biomedical literature. *J Cheminf* 2015;7:1–9. doi: 10.1186/1758-2946-7-S1-S11 PMC4331687 25810768

[43] Wei CH , Allot A, Leaman R et al. PubTator central: automated concept annotation for biomedical full text articles. *Nucleic Acids Res* 2019;47:W587–W593. doi: 10.1093/nar/gkz389 31114887 PMC6602571

[44] Mork JG , Jimeno-Yepes A, and Aronson AR. The NLM medical text indexer system for indexing biomedical literature. In: *Proceedings of the first Workshop on Bio-Medical Semantic Indexing and Question Answering, a Post-Conference Workshop of Conference and Labs of the Evaluation Forum 2013 (CLEF 2013)*. CEUR Workshop Proceedings, Valencia, Spain, September 27, 2013.

[45] Luo ZH , Shi MW, Yang Z et al. pyMeSHSim: an integrative python package for biomedical named entity recognition, normalization, and comparison of MeSH terms. *BMC Bioinf* 2020;21:1–14. doi: 10.1186/s12859-020-03583-6 PMC7301509 32552728

[46] Peng S , You R, Wang H et al. DeepMeSH: deep semantic representation for improving large-scale MeSH indexing. *Bioinformatics* 2016;32:i70–i79. doi: 10.1093/bioinformatics/btw294 27307646 PMC4908368

[47] Tsoumakas G , and Vlahavas I. Random k-labelsets: an ensemble method for multilabel classification. In: *Proceedings of European Conference on Machine Learning*. pp. 406–17. Berlin and Heidelberg: Springer, 2007.

[48] Khushaba RN , Al-Ani A, Al-Jumaily A. Feature subset selection using differential evolution and a statistical repair mechanism. *Expert Syst Appl* 2011;38:11515–26. doi: 10.1016/j.eswa.2011.03.028

[49] Mikolov T , Chen K, Corrado G et al. Efficient estimation of word representations in vector space. *arXiv preprint, arXiv:1301.3781*. 2013.

[50] Pennington J , Socher R, and Manning CD. Glove: global vectors for word representation. In: *Proceedings of the 2014 Conference on Empirical Methods in Natural Language Processing*, pp. 1532–43. Doha: Association for Computational Linguistics, 2014.

[51] Zhu Y , Kiros R, Zemel R et al. Aligning books and movies: Towards story-like visual explanations by watching movies and reading books. In: *Proceedings of the IEEE International Conference on Computer Vision*. pp. 19–27. NW Washington, DC: IEEE Computer Society, 2015.

[52] Liu Y , Ott M, Goyal N et al. Roberta: A robustly optimized Bert pretraining approach. *arXiv preprint, arXiv:1907.11692*. 2019.

[53] Tsoumakas G , Katakis I. Multi-label classification: an overview international journal of data warehousing and mining. *Int J Data Warehous Min* 2007;3. doi: 10.4018/jdwm.2007070101

[54] Hsu CW , Chang CC, Lin CJ. A Practical Guide to Support Vector Classification. 2003. https://www.csie.ntu.edu.tw/~cjlin/papers/guide/guide.pdf (2 December 2022, date last accessed).

[55] Hsu CW , Lin CJ. A comparison of methods for multiclass support vector machines. *IEEE Trans Neural Netw* 2002;13:415–25. doi: 10.1109/72.991427 18244442

[56] Du J , Chen Q, Peng Y et al. ML-net: multi-label classification of biomedical texts with deep neural networks. *J Am Med Inform Assoc* 2019;26:1279–85. doi: 10.1093/jamia/ocz085 31233120 PMC7647240

[57] Fang L , and Wang K. Team Bioformer at BioCreative VII LitCovid Track: multic-label topic classification for COVID-19 literature with a compact BERT model. In: *Proceedings of the 7th BioCreative Challenge Evaluation Workshop*, USA: BioCreative, Cecilia Arighi, University of Delaware, pp. 272–74. 2021.

[58] Baker S , Silins I, Guo Y et al. Automatic semantic classification of scientific literature according to the hallmarks of cancer. *Bioinformatics* 2016;32:432–40. doi: 10.1093/bioinformatics/btv585 26454282

[59] Weber L , Sänger M, Münchmeyer J et al. HunFlair: an easy-to-use tool for state-of-the-art biomedical named entity recognition. *Bioinformatics* 2021;37:2792–94. doi: 10.1093/bioinformatics/btab042 33508086 PMC8428609

[60] Wang LL , Lo L, Chandrasekhar Y et al. CORD-19: The COVID-19 open research dataset. In: *Proceedings of the 1st Workshop on NLP for COVID-19 at ACL 2020*. Association for Computational Linguistics, Online. 2020.

[61] An X , Zhang M, Xu S. An active learning based approach for screening scholarly articles about the origins of SARS-CoV-2. *PLoS One* 2022;17:e0273725. doi: 10.1371/journal.pone.0273725 PMC9480989 36112646

[62] Munkhdalai T , Faruqui M, Gopal S. Leave no context behind: efficient infinite context transformers with infini-attention. *arXiv preprint, arXiv*:2404.07143. 2024.

